# Updating “Stroke 1‐2‐0” by Extending Recognition to Posterior‐Circulation Stroke and Reinforcing Rapid Action in China

**DOI:** 10.1002/cns.70764

**Published:** 2026-01-27

**Authors:** Jing Zhao, Qiuhong Ji, Xunming Ji, Renyu Liu

**Affiliations:** ^1^ Department of Neurology Minhang Hospital Affiliated to Fudan University Shanghai China; ^2^ Department of Neurology Affiliated Hospital of Nantong University Nantong China; ^3^ Department of Neurosurgery Xuanwu Hospital, Capital Medical University Beijing China; ^4^ Departments of Anesthesiology and Critical Care, and Neurology Perelman School of Medicine at the University of Pennsylvania Philadelphia Pennsylvania USA

## Abstract

“Stroke 1‐2‐0” is a Chinese numeric mnemonic linking face drooping, arm weakness, and speech difficulty to calling emergency services (1‐2‐0). We propose an enhanced repeated version—“Stroke 1‐2‐0, Stroke 1‐2‐0”—that adds visual impairment and imbalance to better detect posterior‐circulation strokes, while repetition may boost recall and stresses zero delay in seeking help.

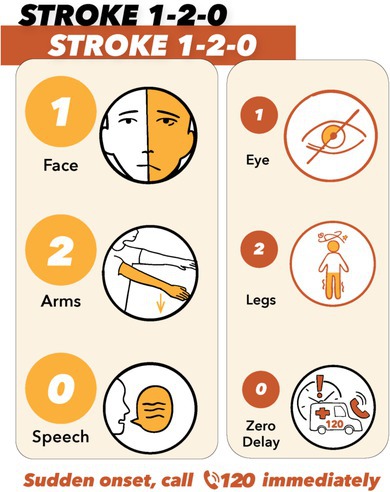

International evidence consistently shows persistent global deficits in stroke awareness and timely action, with many people unable to recognize stroke symptoms or intending to use Emergency Medical Service (EMS) even in high‐income settings. Recent works confirm that recognition of classic symptoms is low to moderate worldwide, and intended responses often remain inappropriate [1, 2, 3]. These gaps are worsened outside English‐speaking regions, where mnemonics such as FAST are difficult to translate with limited linguistic mapping to non‐alphabet‐based languages [4, 5]. Such challenges directly motivated the development of numerical, language‐neutral tools [5, 6]. In 2016, we introduced Stroke 1‐2‐0 as a culturally attuned, number‐based mnemonic for stroke recognition and action in China, where 1‐2‐0 is the emergency medical service (EMS) phone number [5]. In the Stroke 1‐2‐0 system, “1” prompts checking for an uneven face, “2” for weakness in two arms, and “0” for unclear speech—each triggering a call to 1‐2‐0 [5]. The direct linkage of stroke signs and symptoms allowed quick action to call 1‐2‐0.

The program has demonstrated strong public‐health value. A large community study in Shanghai showed significantly reduced prehospital delays, and middle‐school students were able to learn and pass the mnemonic to family members [7, 8]. The effectiveness of Stroke 1‐2‐0 is also demonstrated in primary school students in China [9]. The program has influenced the culture and relevant guidelines to improve stroke care in China [10]. Some proposed a new Stroke 1‐2‐0 based strategy to improve rapid identification and action [11].

Despite the success, Stroke1‐2‐0—like FAST—misses many posterior‐circulation strokes (PCS), which frequently present with a sudden visual disturbance (e.g., monocular or hemifield loss, diplopia) and acute imbalance/dizziness with gait ataxia [12]. These “non‐FAST” symptoms contribute to delayed EMS activation and prolonged door‐to‐needle times. Although BE‐FAST adds “Balance” and “Eye” symptoms, it remains English letter‐based [13].

To address this, this conceptual editorial commentary proposes strengthening the original Stroke 1‐2‐0 by repeating the sequence. The first “1‐2‐0” remains unchanged; the second focuses on PCS:

- 1—One eye or 1 pair of eyes: sudden blurred vision or vision loss It is important to emphasize that multiple visual symptoms may occur and can involve one eye or both eyes.
- 2—Two legs: imbalance or inability to stand steadily.
- 0—Zero delay: call 1‐2‐0 immediately.

A new cartoon (Figure 1) visually distinguishes anterior‐ and posterior‐circulation stroke signs while maintaining a unified teaching script. Although the meanings of “1,” “2,” and “0” differ slightly across the two lines, the overlap in anatomy and behavior reduces potential confusion. For the public, the elements can be taught as a single cue set: ‘1’ for an uneven face or vision problems; ‘2’ for checking two arms for arm weakness or two legs for inability to stand steadily; and ‘0’ for unclear speech or zero delay—call 1‐2‐0 immediately.

**FIGURE 1:**
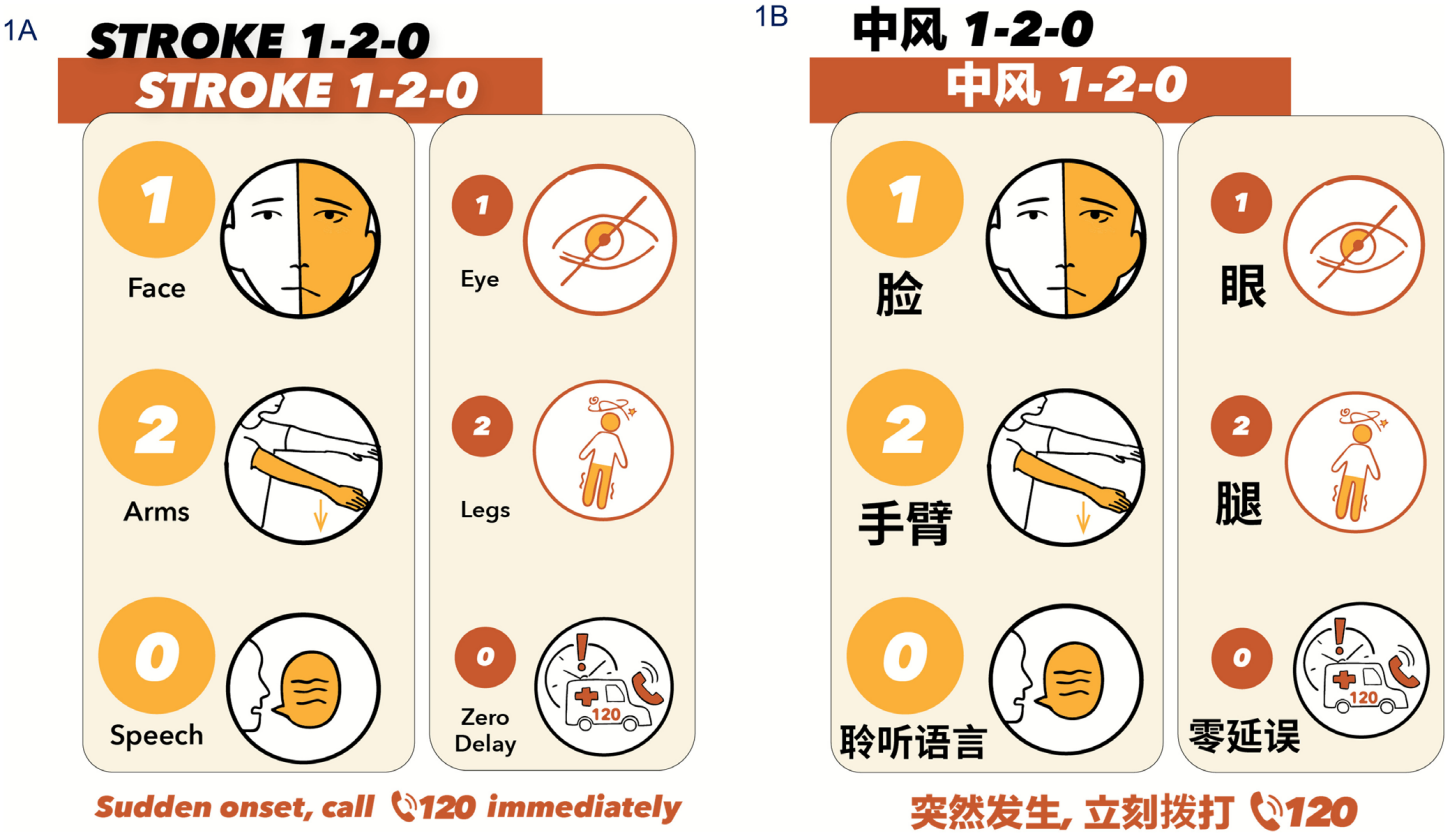
Cartoon of Stroke 1‐2‐0, Stroke 1‐2‐0 in English and Chinese. (A) English version for paper‐review purpose. (B) Chinese version for public education. Because face, arm, and speech signs are the most common, we intentionally enlarged the left‐hand panel. For public education, teach the elements as a unified cue set: “1” = uneven face (left) or eye vision problems (right); “2” = check two arms for one‐arm weakness (left) or two legs for unsteady standing due to dizziness (right); and “0” = unclear speech (left) or zero delay—call 1‐2‐0 immediately (right) if any sign appears suddenly on either side. Cartoon credit to Lilian Liu. Original figure is available upon request from the corresponding author.

This update offers three major benefits: it strengthens recall, expands recognition to PCS, and reinforces stroke action awareness. (1) It reinforces the well‐accepted Stroke 1‐2‐0 program and enhances recall through a simple echo (“Stroke 1‐2‐0, Stroke 1‐2‐0”), Public campaigns should present the update as “the same, then more”—not a replacement, but an augmentation; (2) it expands coverage beyond previous Stroke 1‐2‐0 signs to include key PCS symptoms, and (3) it improves the stroke action awareness—dial 1‐2‐0 immediately when a stroke sign or symptom is spotted. This preserves the recognition speed and cultural resonance of the original number‐based design while broadening sensitivity to strokes that otherwise risk being overlooked. This also aligns well with our recent call to improve stroke action awareness, not just to improve the understanding or knowledge of stroke signs and symptoms by repeating the action of calling 1‐2‐0 after spotting a potential stroke [14].

To address the need for a structured evaluation framework, we outline a staged validation and implementation pathway (Table 1). We propose beginning with a streamlined expert‐acceptance evaluation (a single combined survey with targeted mini‐vignettes) followed by refinement to ensure clarity, safety, action‐specificity (“call 1‐2‐0 now”). Contingent on meeting pre‐set thresholds, we will then conduct a public acceptance test including assessing comprehension, recall, and intention to call 1‐2‐0. Only strategies that pass both the expert and public acceptance phases will be advanced to broaden public education. Throughout, we will engage various professionals (neurology, emergency medicine, EMS/dispatch, nursing, public health, and campaign experts, stroke coordinators, and media partners), seek alignment and support from relevant policymakers, involve stroke survivors, their family members, and community members for survey and workshop design to ensure consistent messaging, sustainable implementation, and rapid scale‐up.

**TABLE 1:** Conceptual evaluation framework for the updated “Stroke 1‐2‐0, Stroke 1‐2‐0 program.”

Stage	Purpose	Population	Key assessment
Expert review and revision	Confirm clarity, recall, and action focus; refine messaging	Stroke clinicians, EMS, public‐health experts	Clear and safe messages; inclusion of posterior‐circulation signs
Public acceptance	Assess understanding, recall, and intended response	General public, caregivers	Comprehension; symptom recall; intention to call 1‐2‐0
Real‐world impact	Measure effect on stroke response and care timelines	EMS systems, hospitals	Onset‐to‐call time; EMS use; onset‐to‐door time
Implementation	Evaluate feasibility and consistency	Dispatch centers, ED, communities	Reach; adherence to messaging; feasibility
Sustainability	Support long‐term adoption	Health authorities, policymakers	Integration into ongoing programs

Abbreviations: ED, emergency department; EMS, emergency medical service.

To ensure sustainable implementation, we propose a multi‐city, stepped‐wedge roll‐out comparing pre‐ vs. post‐ assessment in regions adopting the updated messaging. Primary assessment may include onset‐to‐call time, EMS usage, onset‐to‐door and door‐to‐needle/door‐to‐reperfusion times, and the proportion of PCS among EMS‐identified stroke alerts. Process measures should capture campaign reach, dispatch adherence to updated PCS prompts, and ED triage metrics. Prior community interventions built around the original Stroke 1‐2‐0 have shown associations with shorter delays and improved ambulance usage [8]. Promotion strategies may involve school‐based curricula, targeted social media campaigns, partnerships with community health centers, and alignment with national stroke‐awareness programs. Potential indicators of effectiveness include decreased onset‐to‐call times, higher EMS activation rates, and improved public awareness of posterior‐circulation stroke signs.

“Stroke 1‐2‐0, Stroke 1‐2‐0” (中风1‐2‐0, 中风1‐2‐0) preserves the strengths of our original number‐based stroke mnemonic while extending recognition to posterior‐circulation signs. By keeping the first line unchanged and dedicating the second to sudden onset of blurred vision and/or vision loss or two‐leg imbalance due to dizziness, the update offers an easy‐to‐teach, easy to remember enhancement aligned with the goal of faster EMS activation for every stroke. This commentary does not assess behavioral effectiveness, which will be addressed in future studies guided by the proposed framework.

## Funding

This work was supported by Penn Global at the University of Pennsylvania, 0000000115.

## Conflicts of Interest

The authors declare no conflicts of interest.

## Data Availability

Data sharing not applicable to this article as no datasets were generated or analysed during the current study.
